# Aberrant sparganosis in cat caused by *Spirometra mansoni* (Cestoda: Diphyllobothriidae): a case report

**DOI:** 10.1186/s12917-024-03995-z

**Published:** 2024-04-20

**Authors:** Toshihiro Tokiwa, Momo Fushimi, Shyun Chou, Akemi Yoshida, Kensei Kinoshita, Atsushi Hikima, Taisei Kikuchi, Kiyokazu Ozaki

**Affiliations:** 1 Laboratory of Veterinary Parasitology, Nippon Veterinary and Life Science University, Kyonancho, Musashino, Tokyo Japan; 2 Fushimi Animal Hospital, Hanawa, Mashikomachi, Hagagun, Tochigi Japan; 3 Department of Veterinary Medicine, College of Veterinary Medicine, National Chung Hsing University, Xing Da Road, Taichung, Taiwan; 4 Genomics and Bioenvironmental Science, Frontier Science Research Center, University of Miyazaki, Miyazaki, Japan; 5 Department of Integrated Biosciences, Graduate School of Frontier Sciences, The University of Tokyo, Chiba, Japan; 6 Department of Infectious Diseases, Faculty of Medicine, University of Miyazaki, Miyazaki, Japan; 7 Laboratory of Pathology, Setsunan University, Nagaotohgecho, Hirakata, Osaka, Japan

**Keywords:** Domestic cat, *Felis silvestris catus*, Next-generation sequencing, Paraffin-embedded tissue, Sequence analysis, *Spirometra mansoni*

## Abstract

**Background:**

Sparganosis is a rare zoonotic disease caused by plerocercoid larvae of the genera *Spirometra* or *Sparganum* (Cestoda: Diphyllobothriidae). The larvae of *Spirometra* generally do not undergo asexual reproduction, whereas those of *Sparganum* can induce proliferative lesions in infected tissues. This paper presents an unusual case of proliferative sparganosis due to infection with *Spirometra mansoni* in a cat, normally considered a definitive host of the species.

**Case presentation:**

A 9-year-old male domestic cat was presented with a mass on the right side of the face that underwent progressive enlargement for 1 month. The morphological and histopathological examinations revealed multiple asexual proliferative cestode larvae in the lesions, suggestive of proliferative sparganosis. Next-generation sequencing analysis of formalin-fixed and paraffin-embedded specimens of surgically excised tissue indicated that the worm was *Spirometra mansoni.*

**Conclusion:**

Although *S. mansoni* a common tapeworm species found in the small intestine of domestic cats and dogs in Japan, proliferative sparganosis is extremely rare. This is the first confirmed case of proliferative sparganosis due to infection with *S. mansoni* in cat.

## Introduction

Sparganosis is caused by the infection of the spargana of *Spirometra* spp. and *Sparganum proliferum* [1]. The spargana of *Spirometra* generally do not undergo asexual reproduction, whereas those of *S. proliferum* can induce proliferative lesions in infected tissues, with multiple larvae present at a single site [1, 2]. Sparganosis in cat is considered as rare and is mostly non-proliferative, and is caused by infection with *Spirometra* spp. In the present case, live aberrant spargana were detected in the subcutaneous tissue of a cat, which morphologically indicated proliferative sparganosis caused by *S. proliferum*. However, it was confirmed by next-generation sequencing as proliferative sparganosis incriminated to the spargana of *S. mansoni*. The present case represents the first confirmed case of proliferative sparganosis in a cat caused by *S. mansoni.*

## Case report

A 9-year-old neutered male cat was presented in November 2020 with a 1-month history of soft tissue swelling on the right side of the face. The lesion had an inflamed surface and produced a white discharge when incised with a needle. The cat was treated with an antibiotic (amoxicillin, 20 mg/kg, BID, for 8 d) and corticosteroids (oral, 0.3 mg/kg, BID, for 8 d).

**Fig. 1:**
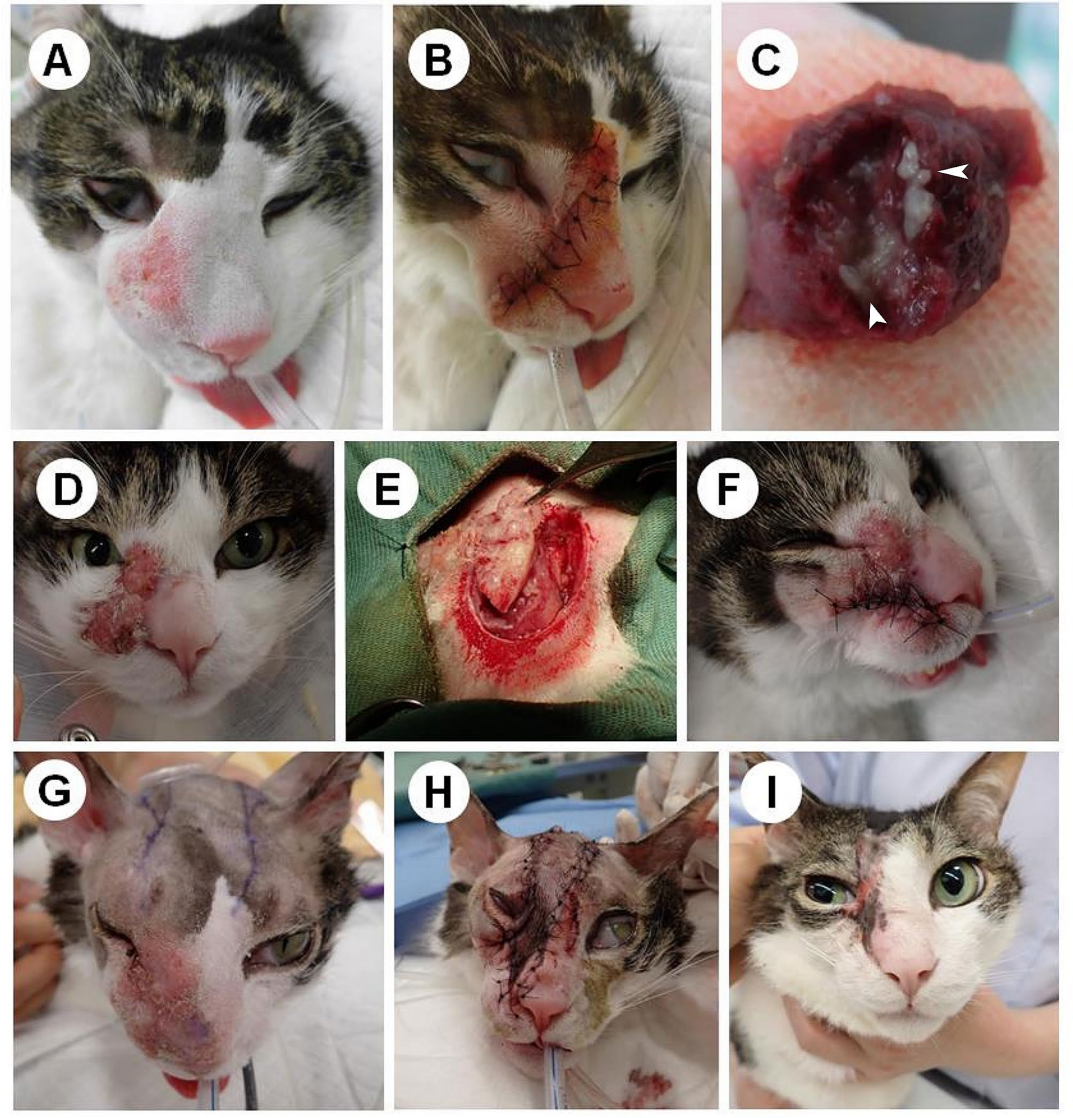
Treatment process for aberrant feline sparganosis. Before (**A**) and after (**B**) treatment in January 2021, and the excised tissue (**C**) showed white discoloration (arrowheads). In January 2022, the postoperative area continues to exhibit itchiness, hair loss, and significant swelling (**D**), and skin biopsy was performed (**E, F**). In April 2022, the affected area exhibits futher swelling (**G**). After excision of the lesion, skin flap reconstruction was performed (**H**). Two weeks after surgery (**I**)

In January 2021, the cat was brought back to the hospital owing to increased swelling of the lesion (Fig. 1A). The lesion was surgically removed under general anesthesia (Fig. 1B). Complete excision of the mass was challenging because of limited skin availability, and a portion of the lesion remained in the surrounding area. White foreign structures were observed on the excised tissue surfaces (Fig. 1C). Histopathological examination revealed multiple worm sections, leading to the diagnosis of helminthic infection. The cat was administered with the Broadline® spot-on solution (Merial, Japan), a combination product comprising praziquantel, eprinomectin, fipronil, (S)-methoprene four times per month. However, the cat continued to scratch its face. An Elizabethan collar was fitted, and corticosteroids (1 mg /kg, SID, 4 d, followed by 0.6 mg/kg, SID, for 6 d) were administered. In April 2021, the skin lesions recurred and became pruritic. Corticosteroids (1 mg/kg, SID, PO) were administered for 1 year necessarily to relieve itching.

**Fig. 2:**
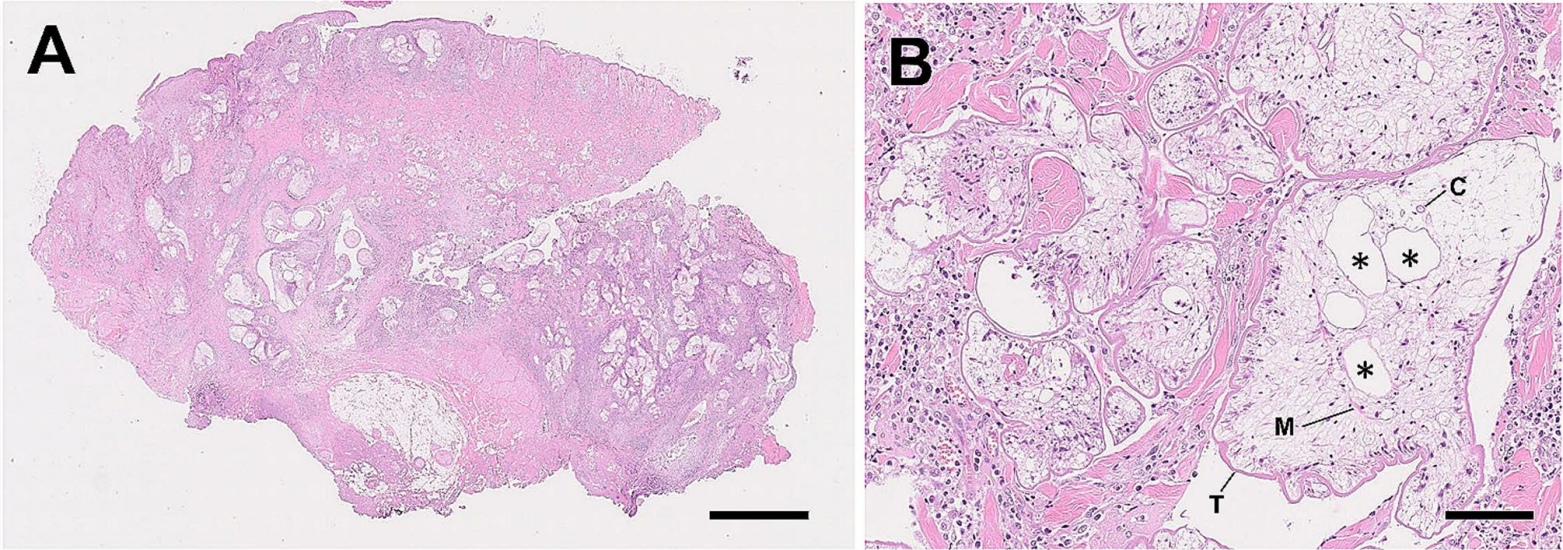
Microscopic images of biopsy tissue from the subcutaneous mass on the face and plerocercoid larvae observed in excised tissues in January 2022. **A**) Numerous, slightly acidic, unequal in size, round to polymorphous sparganum present between the epidermis (upper) and the musculature (lower). Scale bar = 2.5 mm. **B**) External microvilli tegumentary layer (T), calcareous corpuscle (C), enlarged excretory canals (asterisks), and musculature (M) in the parenchyma. Scale bar = 100 μm

In January 2022, the lesions had spread further (Fig. 1D). Biopsy tissues from the skin lesions (Fig. 1E and F) were fixed in 10% neutral-buffered formalin and processed into paraffin blocks using routine procedures. Paraffin-embedded tissues were cut into 5 μm sections and stained with hematoxylin and eosin stain. Microscopic observation showed numerous worm sections of various sizes and shapes in the subcutaneous tissue, extending into the intracutaneous muscles and dermis just below the epidermis (Fig. 2A). The worms were surrounded by macrophages, neutrophils, lymphocytes, and plasma cells, resulting in granulomatous inflammation. These worms were characterized as acephalic without a rostellum and suckers, having tegument bearing microtriches, parenchyma with numerous calcareous bodies, enlarged excretory canals within a loose stroma, bilateral symmetry and irregularly developed musculature (Fig. 2B). Based on these, the worms were identified as proliferative spargana of *S. proliferum*.

**Fig. 3:**
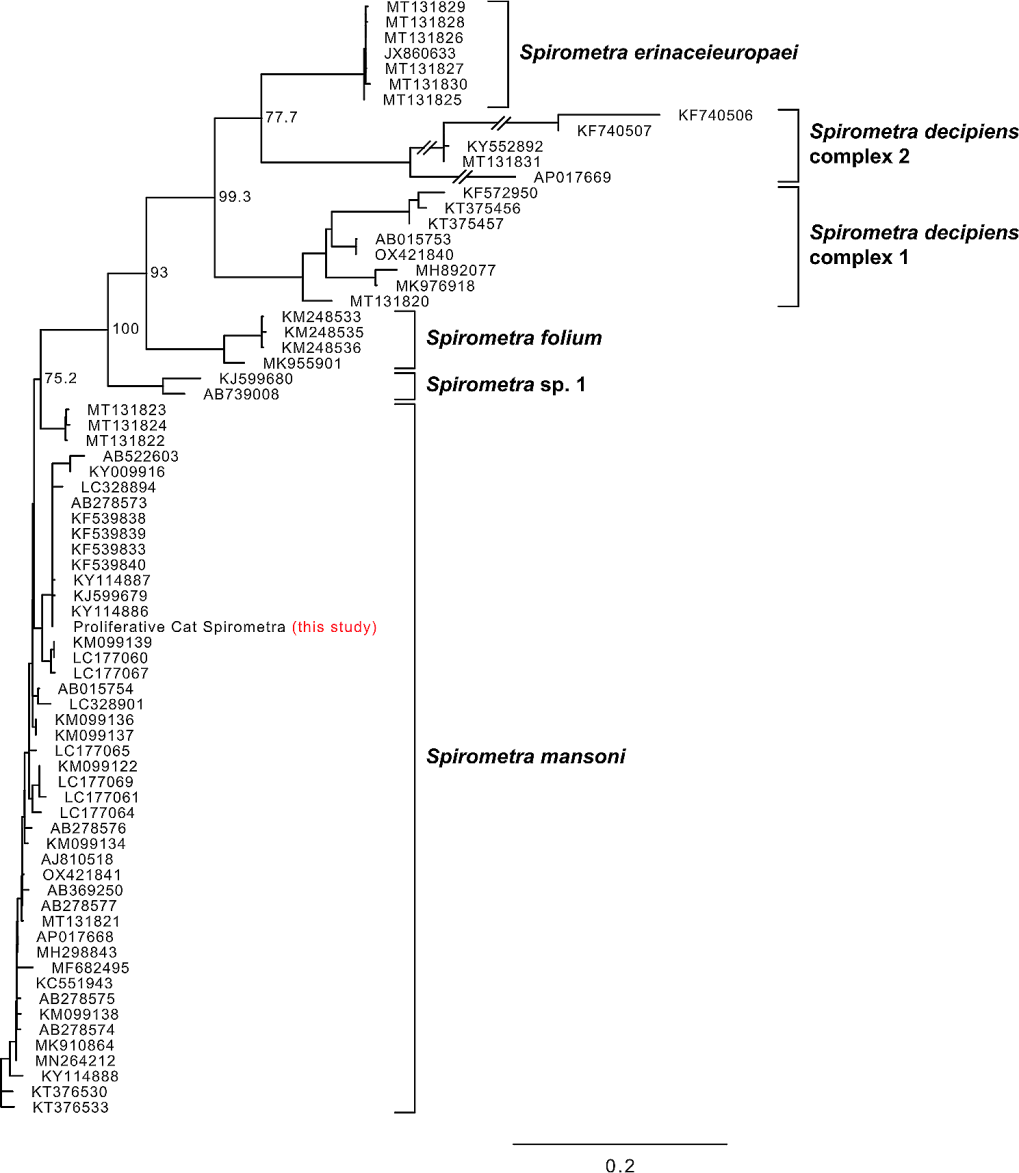
A mid-point rooted phylogenetic tree of cytochrome *c* oxidase subunit I gene of *Spirometra* species. The mitochondrial sequence obtained in this study and 71 sequences retrieved from GenBank/EMBL-Bank/DDBJ were aligned using MAFFT with the L-INS-i option. A maximum likelihood tree was then generated using IQ-TREE with TPM3u + F substitution model. Branch support values were obtained by SH-aLTR test with 1000 replicates and were shown for key blanches in the tree. The scale bar indicates numbers of substitutions per site

DNA was extracted from two 10 μm formalin-fixed paraffin-embedded sections using the GeneRead DNA FFPE kit (Qiagen) according to the manufacturer’s protocol. A total of 420 and 1,460 ng DNA was obtained from the two sections with A260/A280 (absorbance at 260 nm and 280 nm) ratios of 1.92 and 1.86, respectively. An Illumina sequencing library was prepared from each extracted DNA sample (100 ng) using an Illumina DNA Library Prep Kit (Illumina) following the manufacturer’s protocol. Those libraries were sequenced on MiSeq using the MiSeq reagent kit v3 producing 300-bp paired-end reads. The resulting 4.2 and 3.9 Gbp raw sequencing data was deposited under DDBJ BioProject no. PRJDB16823. Metagenomic species profiling of the total reads was performed using CCMetagen [3]. The results indicated that ~ 47% of the reads were from Cestoda, ~ 38% were from Mammalia (mainly *Felis*), and ~ 17% were from *Drosophila*. Approximately 20% of Cestoda reads were classified at the genus level (*Spirometra*). To identify the species, we extracted mitochondrial reads from the metagenome dataset and constructed mitochondrial genomes using the GetOrganelle pipeline [4]. Those libraries were sequenced on MiSeq using the MiSeq reagent kit v3 producing 300 bp paired-end reads. The circular mitochondrial genome assembly (accession no. ERZ21839573) was 13,643 bp long and showed high similarity (87–99%) to the mitochondrial sequences of *Spirometra* spp. A maximum likelihood tree was generated using the cytochrome *c* oxidase subunit I gene (1,566 bp) of mitochondria with 71 sequences deposited as cytochrome *c* oxidase subunit I gene of *Spirometra* spp. in the sequence repository (Fig. 3). The tree showed six well-defined clusters corresponding to separate species with a clear geographical pattern as described in Kuchta et al. [6]. The mitochondrial sequence obtained in this study was placed in the middle of *S. mansoni* cluster with 100% identity with that from China (accession no. KY114886). The examination results were presented to the owner with the need for additional surgery; however, the owner declined.

The cat was brought back to the hospital on April 16, 2022. The lesion on the right side extended from under the eye to the nose and cheek and spread further to the inner corner of the right eye (Fig. 1G). When the lesion was incised, numerous motile, white, glandular, and irregularly shaped spargana were observed in the subcutaneous tissue and on the skin surface. The lesion was thoroughly excised, and skin flap surgery was performed on the head (Fig. 1H). Intraoperatively, a five-fold dilution of praziquantel was administered into the normal subcutaneous tissue after removing the lesion for several minutes. On April 30, 2022, the sutures were removed (Fig. 1I). Praziquantel (0.5 ml/kg) was administered subcutaneously on the scapula, with two doses administered on April 30, 2022 and 2 months later. As of October 2023, there was no recurrence of the lesion at the surgical site.

## Discussion and conclusion

The taxonomic status of *Spirometra* species remains controversial [5]. Recent studies have suggested the reclassification of *Spirometra* into six species/lineages: *S. erinaceieuropaei* mainly in Europe; *S. decipiens* complexes 1 and 2 mainly in America; *S. mansoni* in the Eurasian and Oceanic regions; *S. folium* in Africa; and *S. asiana* mainly in Japan and Korea [6, 7]. According to this classification, the species detected in the present case was identified as *S. mansoni*, not *S. erinaceieuropaei*.

Domestic cats can act as final hosts as well as accidental second intermediate or paratenic hosts for *Spirometra* species [8]. Although the site of predilection for the adult worms is the small intestine of cats, but the detection of spargana is rare. A 7-year-old cat, which had been purchased in Cambodia, raised in Taiwan for three years, and later cared for in the Unites States, was found to have ribbon-like spargana measuring about 3 cm in length in the mass of stomach wall [9]. In a survey conducted from 1982 to 1984 in Hyogo, Japan, sparganum were observed in the subcutaneous tissues or body cavities of only 10 of the 1,880 free-roaming cats [10]. The non-proliferative sparganum in these cats were ribbon-like, measured 11.5 cm (2.0–28.0 cm) in length, and had distinct morphology from that of the sparganum detected in our feline case. Reports of proliferative sparganosis in cats are extremely rare. A case of proliferative sparganum was detected in the internal organs of a 6-year-old domestic cat in Florida [11]. In two cases of 8-year-old cats reported in Georgia [12], swelling was observed in the lumbar and ventral neck area, and the proliferative sparganum, measuring 1–1.5 mm in diameter, was found in the cyst masses of the lesions. Except for the site of infection, these characteristics are similar to those of the present case; however, the causative species is unclear. Therefore, the present case represents the first confirmed case of proliferative sparganosis in a cat caused by *S. mansoni*.

The cause of sparganosis in cats remains unclear. As the cat in the present case had access to the outdoor environment, it might have consumed water contaminated with infected copepods or ingested secondary intermediate hosts, such as snakes and frogs [8]. Treatment of non-proliferative sparganosis caused by *Spirometra* involves the surgical removal of ribbon-like plerocercoids from a mobile or regressing mass. In some human cases, praziquantel or mebendazole may also be administered to treat the remaining parasites [1]. In the present case, extracting only the sparganum was challenging because of the intricate infestation within the subcutaneous and muscle tissues. Therefore, the affected tissues were completely excised, which did not result in recurrence.

In conclusion, this case represents an aberrant form of feline sparganosis caused by *Spirometra* species. Veterinary clinicians should consider sparganosis as a potential diagnosis when encountering subcutaneous masses in cats.

## Data Availability

All data generated or analyzed during this study are included in this published article.
